# Dependence of Quantized Hall Effect Breakdown Voltage on Magnetic Field and Current

**DOI:** 10.6028/jres.098.028

**Published:** 1993

**Authors:** M. E. Cage

**Affiliations:** National Institute of Standards and Technology, Gaithersburg, MD 20899-0001

**Keywords:** breakdown, inter-Landau level scattering, quantized dissipation, quantized voltage states, quantum Hall effect, two-dimensional electron gas

## Abstract

When large currents are passed through a high-quality quantized Hall resistance device the voltage drop along the device is observed to assume discrete, quantized states if the voltage is plotted versus the magnetic field. These quantized dissipative voltage states are interpreted as occurring when electrons are excited to higher Landau levels and then return to the original Landau level. The quantization is found to be, in general, both a function of magnetic field and current. Consequently, it can be more difficult to verify and determine dissipative voltage quantization than previously suspected.

## 1. Introduction

The integer quantum Hall effect [1] occurs when current is passed through a two-dimensional electron gas (2DEG) formed in a semiconductor device which is cooled to very low temperatures in the presence of a large magnetic field. The Hall resistance *R*_H_ of the *i*th plateau of a fully quantized 2DEG assumes the values *R*_H_(*i*)=*h*/(*e*^2^*i*), where *h* is the Planck constant, *e* is the elementary charge, and *i* is an integer. In high-quality devices the current flow within the 2DEG is nearly dissipationless in the plateau regions for currents around 25 μA. At high currents, however, energy dissipation can suddenly appear in these devices [2,3]. This is called breakdown of the quantum Hall effect.

The dissipative breakdown voltage *V_x_* can be detected by measuring voltage differences between potential probes placed on either side of the device in the direction of current flow. Cage et al. [3] had found that there is a distinct set of dissipative *V_x_* states in wide samples, with transient switching observed on microsecond time scales among those states. Bliek et al. [4] proposed the existence of a new quantum effect to explain the breakdown structures in their curves of *V_x_* versus magnetic field for samples with narrow constrictions. Their phenomenological model presumed that the structures were quantized in resistance, rather than voltage. Cage et al. [5] then found that, in wide samples, the distinct states are quantized in voltage. Hein et al. [6] have now observed dissipative voltages during breakdown of the quantum Hall effect in wide samples, but did not confirm that these voltage states are quantized. We show in this paper that the voltage is indeed quantized, but that the quantization is more complicated than previously suspected because, in general, it is a function of both the magnetic field and the current. Some of the data presented here were described with less detail in an earlier paper [7].

## 2. Experiment

### 2.1 Sample

**Fig. 1:**
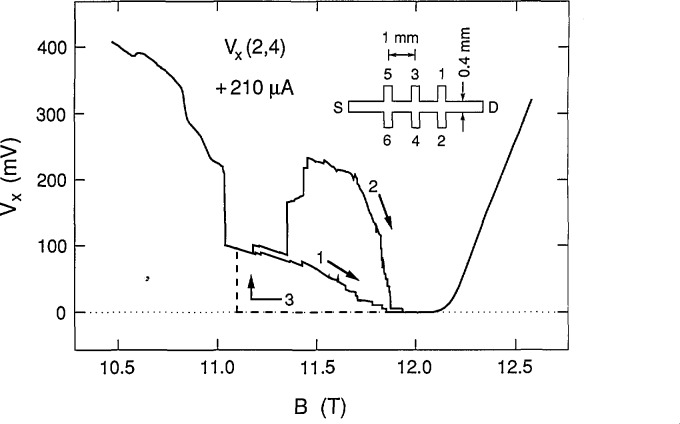
Sweeps of *V_x_*(2,4) versus *B* for the *i* = 2 plateau at +210 μA and 1.3 K. Two of the sweeps (paths 1 and 2) are in the increasing *B* direction. The dashed line (path 3) shows hysteresis for a sweep in the decreasing *B* direction. The inset displays the sample geometry.

Our sample is a GaAs/Al*_x_*Ga_1−_*_x_*As heterostructure grown by molecular beam epitaxy with *x* = 0.29. It is designated as GaAs(7), has a zero magnetic field mobility of 100,000 cm^2^/(V·s) at 4.2 K, exhibits excellent integral quantum Hall effect properties, and is the device most frequently used as the United States resistance standard. The inset of Fig. 1 shows the geometry of this sample. It is 4.6 mm long and 0.4 mm wide. The two outer Hall potential probe pairs are displaced from the central pair by ± 1 mm. The magnetic field is perpendicular to the sample; its direction is such that probes 2, 4, and 6 are near the source potential S, which is grounded. Probes 1, 3, and 5 are near the potential of the drain D. The dissipative voltages *V_x_* for this paper were measured between potential probe pair 2 and 4, hereafter denoted as *V_x_*(2,4) ≡ *V_x_*(2) − *V_x_*(4).

### 2.2 Data

Figure 1 shows sweeps of *V_x_*(2,4) versus the magnetic field *B* for the *i* = 2 (12,906.4 Ω) quantized Hall resistance plateau at a temperature of 1.3 K and a current, *I*, of +210 μA, where positive current corresponds to electrons entering the source and exiting the drain. This current is approaching the 230 μA critical current value for this plateau at which *V_x_* never reaches zero for these particular potential probes. One of two distinct paths always occurred for positive current when magnetic field sweeps were made in the direction of increasing *B*. Those distinct paths are labeled 1 and 2 in the figure. This path “bifurcation” is unusual. It occurred only for the *V_x_*(2,4) probe pair at positive current, and only for the *i* = 2 plateau. A pronounced hysteresis was observed when magnetic field sweeps were made in the opposite direction; this path is indicated by the dashed line, labeled 3. The dashed-line curve was repeatable for all sweeps with decreasing *B*, varying only slightly for the value of *B* at which *V_x_* again rose to path 1. The fact that *V_x_* is zero over such a large magnetic field region for path 3 indicates the existence of a dissipationless state between 11.2–12.2 T.

**Fig. 2:**
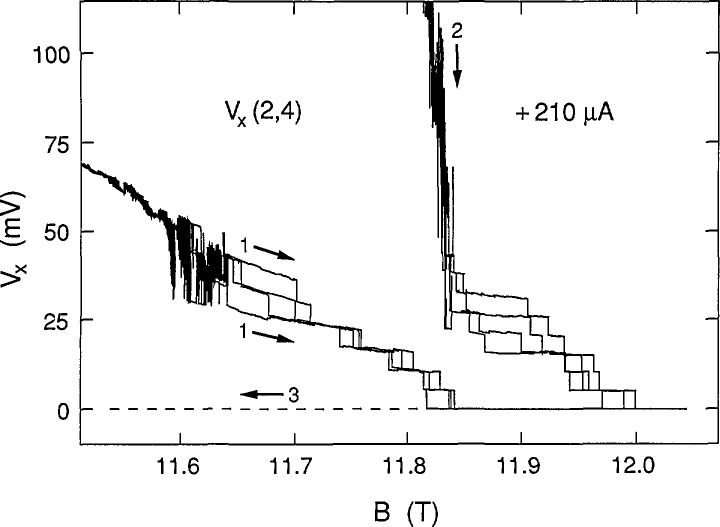
Eight sweeps of *V_x_*(2,4) versus *B* at +210 μA for paths 1 and 2, plus a path 3 sweep.

Figure 2 shows eight consecutive sweeps of *V_x_*(2,4) versus an increasing B over a magnified region on the low magnetic field side of *V_x_* minimum at +210 μA. Four of the sweeps happened to be along path 1, forming one family of curves. Another family was generated by the four sweeps over path 2. A ninth sweep is shown for decreasing *B* along path 3. The data clearly show discrete, well-defined voltage states, with switching between states. The voltage states often have slopes which depend on the magnitude of *B*. Individual sweeps are not identified in the figure because the magnetic field values at which the states switch have no correlation with sweep number.

**Fig. 3:**
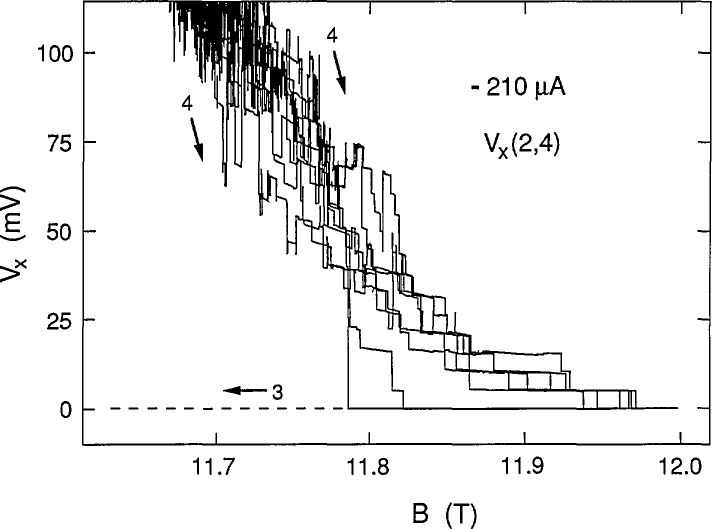
Eight sweeps of *V_x_*(2,4) versus *B* at −210 μA for path 4, and a path 3 sweep.

Figure 3 shows eight consecutive sweeps of *V_x_*(2,4) for increasing *B* at −210 μA. No bifurcation was observed for such sweeps. The family of curves is labeled path 4 in the figure. The curves lie between those of paths 1 arid 2 at +210 μA. Curves for decreasing *B* always followed the dashed line of path 3.

**Fig. 4:**
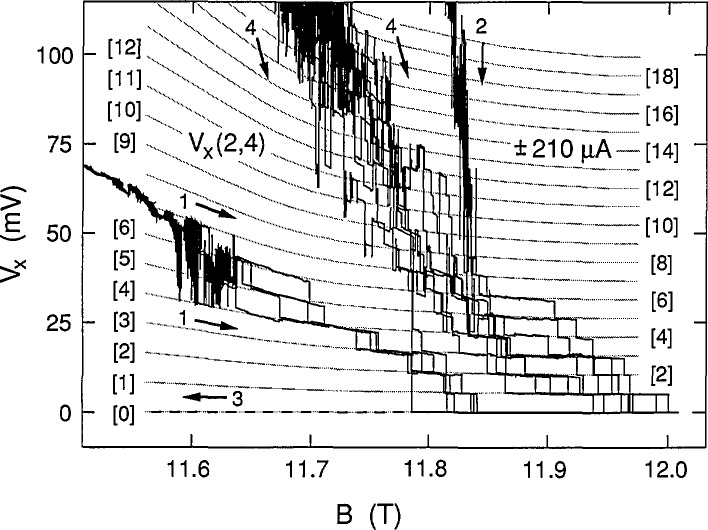
Combination of the data in Figs. 2 and 3 at ±210 μA. A family of 20 shaded curves is fitted to these data. Refer to Sec. 2.2 for an explanation of how the shaded curves were generated. The voltage quantization numbers are shown in brackets.

The data of Figs. 2 and 3 are combined in Fig. 4 to show the 16 consecutive sweeps for increasing *B* at ±210 μA and the two identical sweeps for decreasing *B*. Nothing is unique about these sweeps. Additional sweeps could have been displayed, but at the expense of reducing the overall clarity.

We next demonstrate that the discrete voltage states of Fig. 4 are quantized, and that this quantization is a function of magnetic field. This is done by drawing a family of 20 shaded curves through the data in Fig. 4. The curves have equal (quantized) voltage separations at each value of magnetic field. The quantized voltage separations are, however, allowed to vary with *B* in order to obtain the best fit to the data. The family of curves was generated by first drawing a set of 20 equally-spaced vertical points at a particular value of *B*. The lowest point of the vertical set was constrained to be at 0.0 mV because *V_x_* is always zero in the dashed-line sweep of path 3, which indicates that a dissipationless state exists over the magnetic field region of this figure. The spacing between the 19 other vertical points was then varied to obtain the best fit with uniform (equal) voltage intervals. This procedure was repeated for approximately 30 other values of 5. Finally, a family of 20 smooth shaded curves was drawn through the corresponding points of every vertical set. The 20 shaded curves, which correspond to a *V_x_*=0.0 mV ground state and 19 excited states, are labeled in brackets as [0] through [19]. The voltage separation (quantization) varies between 5.22 and 7.85 mV over the magnetic field range of this figure.

The breakdown activity shown in Fig. 4 is confined to the region between, but not including, the Hall probe pairs 1,2 and 3,4 of Fig. 1. This was demonstrated by measuring the quantum Hall voltages of both Hall probe pairs at this current. The resulting curves of both probe pairs had structures with deviations of only about ±0.1 mV from the expected ±2,710.3 mV quantum Hall voltage over the plateau region, and therefore were horizontal, straight lines when plotted to the same resolution as in Fig. 4. In addition, the *V_x_* signals were the same on both sides of the sample for probe pairs 1,3 and 2,4.

**Fig. 5:**
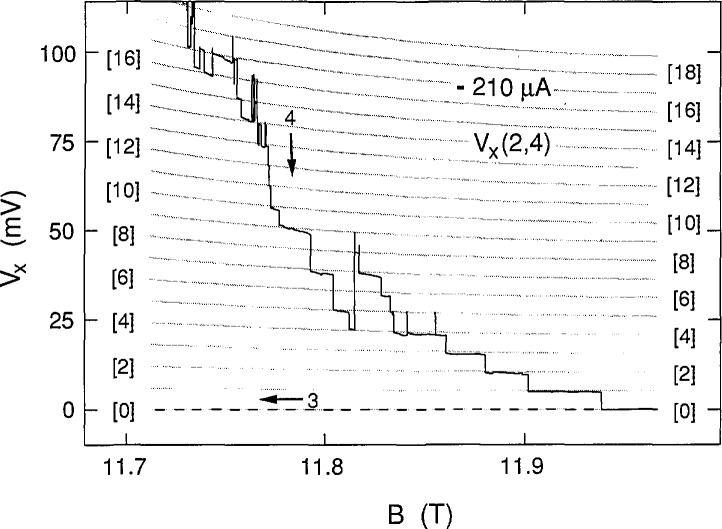
One of the *V_x_*(2,4) versus *B* path 4 sweeps shown in Fig. 4.

The higher-lying excited states are difficult to see in the multiple sweeps of Fig. 4 because of switching between states. Figure 5, therefore, shows one of those sweeps along path 4 at −210 μA. It is remarkable that the higher-lying states are just as well-quantized (i.e., well-fitted by the shaded curves) as the lower-lying states. The quantization is by no means perfect. Deviations from the shaded curves do occur, but the overall trend is clear.

### 2.3 Histograms

**Fig. 6:**
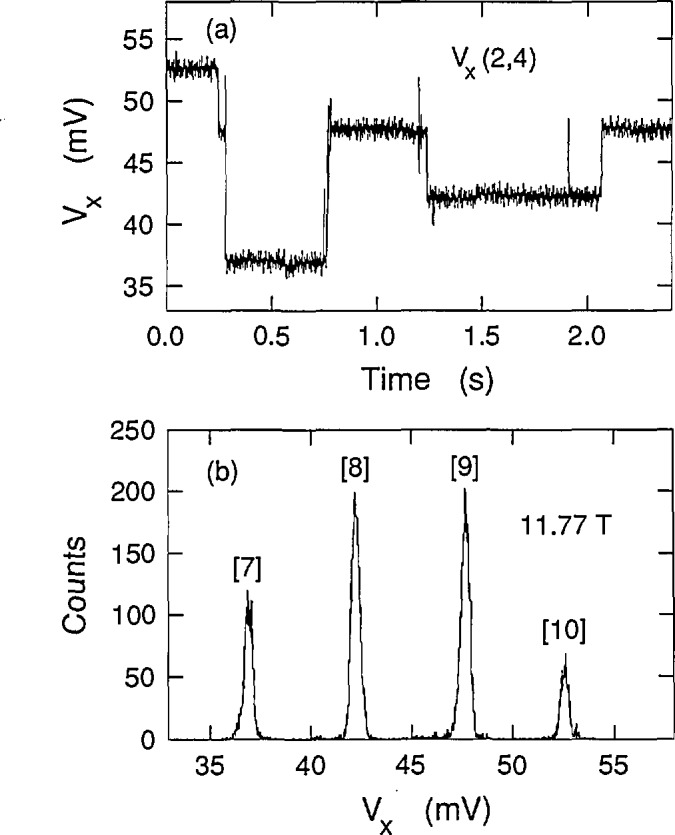
Histogram for a path 4 sweep at 11.77 T. Section 2.3 explains how the histogram was obtained.

**Fig. 7:**
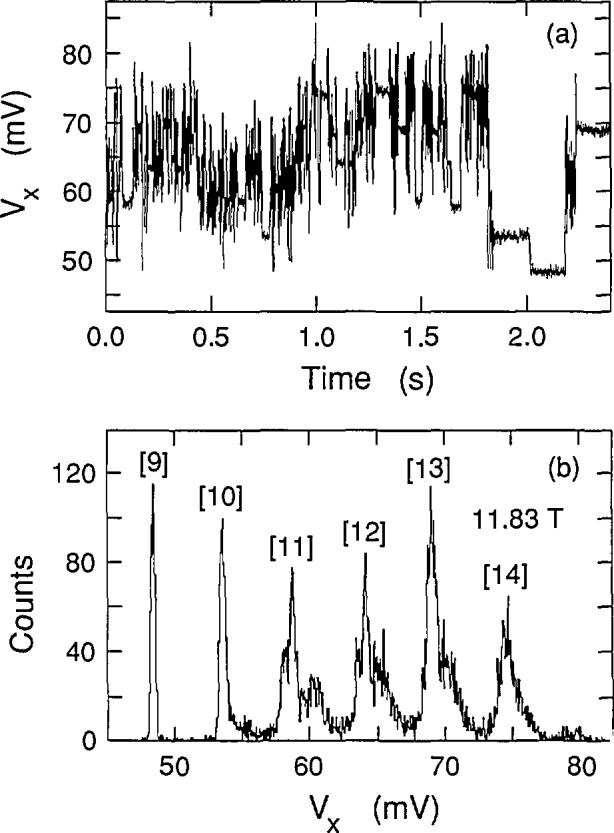
Histogram for a path 2 sweep at +210 μA and 11.83 T.

Cage et al. [8] and Hein et al. [6] have seen that the *V_x_* signal can sometimes be time-averages of two or more discrete dc voltage levels in which only one level is occupied at a time, but where switching occurs between the levels. Therefore, histograms were made to ensure that the signals in Fig. 4 were not time-averages of several levels. Each histogram consisted of 16,000 measurements of the *V_x_* signal in a 2.4 s sampling period. Figure 6(a) shows the time-dependence of one such sampling period for a path 4 sweep at 11.77 T; Fig. 6(b) shows the associated histogram. It is referred to as a histogram, rather than a spectrum, because the areas under the peaks do not correspond to the excitation probabilities. One would have to accumulate many histograms to ascertain the excitation probabilities. For example, peaks corresponding to quantum states 7 through 10 appear in Fig. 6, while other histograms at 11.77 T had missing peaks or additional peaks. These histograms never yielded any voltage states other than the ones which appear in Fig. 4. Figure 7(b) shows another histogram for a path 2 sweep at 11.83 T. The time-dependence of that histogram, shown in Fig. 7(a), suggests several discrete voltage states interspersed with noise, but the histogram is actually composed of voltage quantum states 9 through 14. Histograms of all other high-speed measurements also indicated that there are no subdivisions of the displayed states in Fig. 4.

### 2.4 Other Currents

**Fig. 8:**
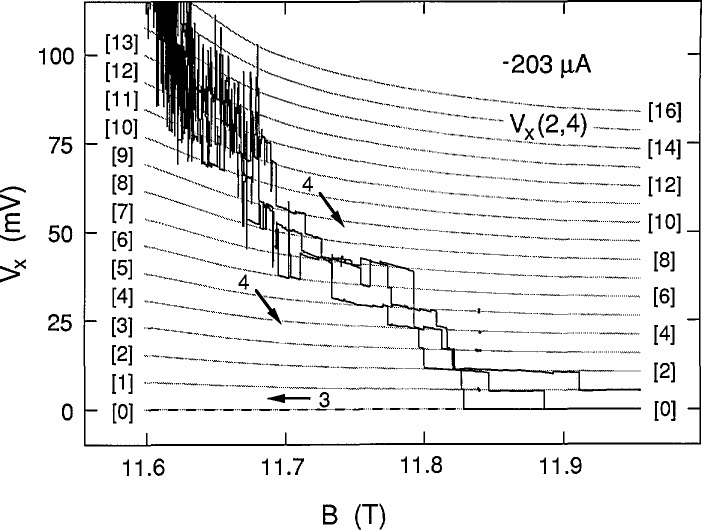
Three sweeps of *V_x_*(2,4) versus *B* at −203 μA for path 4, and a path 3 sweep. Sec. 2.4 describes the individual data points at 11.84 T. The shaded curves are the same family used in Fig. 4.

We next investigate the effect of changing the sample current. The smallest current for which breakdown structures could be observed was at −203 μA; no structures were observed, however, at + 203 μA. Figure 8 shows data for three successive path 4 sweeps at −203 μA, plus a path 3 sweep. The individual data points displayed near 11.84 T were generated by slowly increasing the magnetic field and selecting data points when the voltage switched to new states. Switching to new states was sometimes induced by momentarily increasing the sample current and then reducing it back to −203 μA. This procedure allowed additional data to be included without sacrificing clarity. Figure 8 also shows 17 shaded curves from the same family used to fit the data displayed in Figs. 4 and 5 at ±210 μA. The excellent fit would suggest that the voltage quantization was a function of magnetic field, but not a function of current. However, it will be seen in Sec. 3.3 that, in general, the voltage quantization is a function of current.

**Fig. 9:**
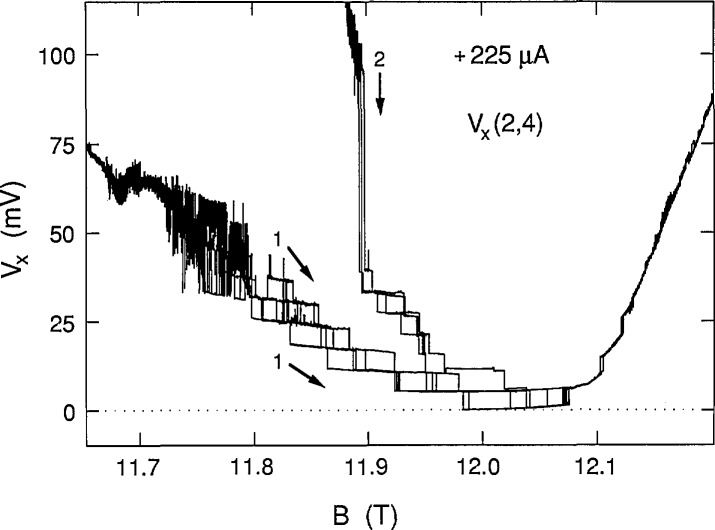
Five sweeps of *V_x_*(2,4) versus *B* at +225 μA for path 1, and four sweeps for path 2.

**Fig. 10:**
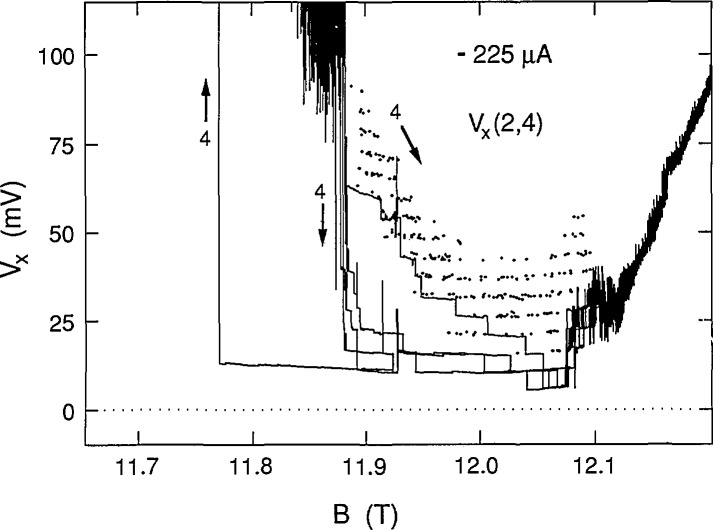
Four sweeps of *V_x_*(2,4) versus *B* at −225 μA for path 4 (along with individual data points), and a sweep along path 4 in the opposite direction.

**Fig. 11:**
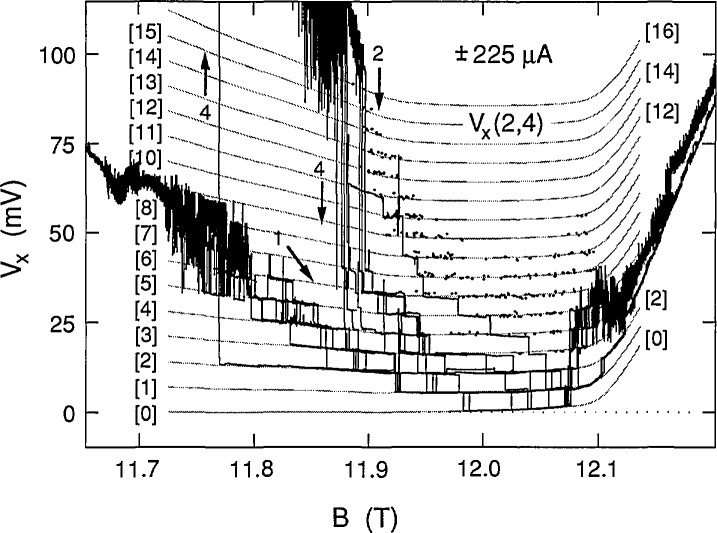
Combination of the data in Figs. 9 and 10 at ±225 μA. A family of 17 shaded curves is fitted to these data.

We chose 225 μA as the highest current because the ground state was still occupied. This current approached the 230 μA critical current value at which *V_x_* was still quantized, but never zero. Figure 9 shows five successive sweeps along path 1 and four successive sweeps for path 2 at +225 μA. Note that there is a gradual deviation from zero voltage on the high magnetic field side of the sweeps. Also, interesting features occur on the high field side of the curves at this current. Figure 10 shows four successive path 4 sweeps for increasing magnetic field at −225 μA, as well as a sweep for decreasing magnetic field. That sweep is also labeled path 4 since it follows much of that path; however, it has hysteresis like that of path 3 sweeps where *V_x_* is zero. Many individual path 4 data points, obtained with increasing magnetic field, are also included in Fig. 10 using the procedure described above. Figure 11 combines the data for the two current directions and displays a family of 17 shaded curves which provide the best fit to the data. The ground state begins deviating from zero at 11.97 T, so the lowest point of each vertical set of 17 points used to generate the 17 shaded curves was no longer constrained to be zero on the right hand side of the figure. This deviation from zero presumably arises from some other dissipative mechanism. It will be shown in Sec. 3.3 that this family of shaded curves for 225 μA is different than that for 203 and 210 μA.

## 3. Interpretation

### 3.1 Microscopic Models

The dissipative voltage states displayed in Figs. 4, 5, 8, and 11 are clearly quantized. We next try to interpret this quantization. Many explanations of breakdown have been proposed. Some mechanisms, such as electron heating instabilities [9] and inhomogeneous resistive channels [10], are inapplicable here since they are classical effects which do not provide quantization. Quantization exists in the quantum Hall effect because the quantized Hall resistance occurs when the conducting electrons in the 2DEG occupy all the allowed states of the lowest Landau levels. It is therefore natural to assume that the quantized dissipation arises from transitions *between* Landau levels.

There are several mechanisms to excite electrons into higher Landau levels that can be considered: (a) the emission of acoustic phonons to conserve energy and momentum, as employed by Heinonen, Taylor, and Girvin [11] and later used in the quasielastic inter-Landau level scattering (QUILLS) model of Eaves and Sheard [12], with refinements and extensions by Cage et al. [8]; (b) Zener tunneling [13]; (c) impurity-assisted resonant tunneling [14]; and (d) transitions between edge states [15,16]. To complicate matters, both bulk and edge states exist at high currents [17]. For bulk transitions, a large electric field (of order 10^6^ V/m) is required somewhere across the width of the sample [8]; sample impurities and inhomogeneities might provide this high local field. The confining potential provides a high electric field for edge states, but if breakdown is due to edge states then it is difficult to understand why breakdown does not always occur at very low currents since there is probably an insignificant change in the slope of the confining potential with current. In addition to the above considerations, one must also take into account the return of the electrons to the ground state via emission of either photons or optical phonons. Furthermore, the dissipative *V_x_* signals are quite large. Most of this dissipation must occur *outside* the breakdown region, otherwise heating effects would depopulate the electron states within the Landau levels and thereby wash-out the quantization.

### 3.2 Simple Model

To avoid controversy about which of those microscopic models [8,11–16] satisfy the above considerations and are appropriate, we use a simple model based on energy conservation arguments, and treat the breakdown region between the Hall probe pairs 1,2 and 3,4 as a black box. We assume that the dissipation arises from transitions in which electrons from the originally full Landau levels are excited to states in higher Landau levels and then return to the lower Landau levels. *V_x_* is then the difference in potential between the initial and final states of the lower Landau level. The electrical energy loss per carrier for *M* Landau level transitions is *Mℏω*_c_, where *ω*_c_=eB/m* is the cyclotron angular frequency, and m* is the reduced mass of the electron (0.068 times the free electron mass in GaAs). The power loss is *IV_x_*. If: (a) the ground state involves several filled Landau levels; (b) only electrons in the highest-filled Landau level undergo transitions; and (c) electrons of both spin sublevels of a Landau level undergo the transitions, then *IV_x_* = *r*(2/*i*)*Mℏω*_c_, where *r* is the total transition rate and *i* is the Hall plateau number. Thus

$$fM=\left(\frac{re}{I}\right)M=\left(\frac{i}{2}\right)\left(\frac{m*}{\hbar }\right)\left(\frac{V_{x}}{B}\right),$$ (1)

where *f* is the ratio of the transition rate *r* within the breakdown region to the rate *I/e* that electrons transit the device; *f* can also be interpreted as the fraction of conducting electrons that undergo transitions. Equation (1) is appropriate for even values of *i*. For odd vales of *i*, the factor *i*/2 should be replaced by the factor *i*.

We associate the quantized values of *M* with the numbers in brackets for the shaded curves in Figs. 4, 5, 8, and 11. *I*, *V_x_*, and *B* are measured quantities, and *i*, m*, and *ℏ* are constants. Therefore, *f* and *r* can be determined from the *V_x_* versus *B* plots and Eq. (1) if *M* is known.

### 3.3 Analysis

**Fig. 12:**
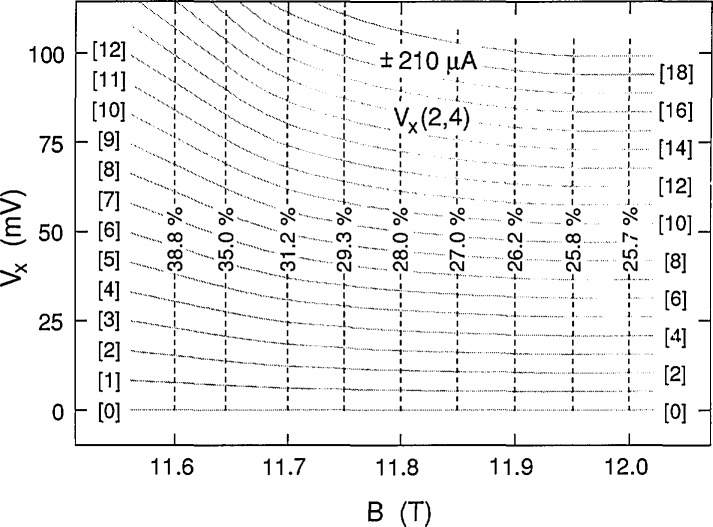
The fractions *f* (expressed as a percentage) of electrons making the Landau level transitions for the 20 shaded curves shown in Fig. 4 at ±210 μA. See Eq. (1) for the definition of *f*. The shaded curves were generated with an accuracy of ~1% and a resolution of ~0.1%.

If *f* and *r* were constant, then *V_x_* ∝ *B* in Eq. (1), but it is clear from Fig. 4 that this is not the case for these data because the slope of *V_x_* versus *B* has the opposite sign. Therefore, both *f* and *r* must vary with magnetic field. The fractions *f* (expressed as a percentage) of electrons that make the transitions in the shaded curves of Fig. 4 were calculated using Eq. (1), and are shown in Fig. 12 at 0.05 T intervals; *f* varies between 25.7% and 38.8%, corresponding to transition rates between 3.4×10^14^/s and 5.1×10^14^/s. Histograms obtained in a previous experiment [5] yielded 26.5% for the value of *f* in the vicinity of 11.75 T, whereas Fig. 12 indicates that *f* is 29.3% at 11.75 T. The apparent discrepancy arises because the position of the *V_x_* minimum varies slightly with *B* on each cool-down. The minimum position was about 0.06 T higher for the present cool-down, giving 27.8% for *f* at 11.81 T, which is in reasonable agreement with the previous result.

Shifted peaks were observed in the previous histograms [5], and were attributed to changes in the *V_x_* zero. There is no evidence for ground state shifts in the present experiment. Instead, the shifted states result from the data deviating from the shaded curves of Fig. 4. This is consistent with having to use peaks from many histograms to obtain the ±0.6% quantization accuracy of the previous experiment [5].

**Fig. 13:**
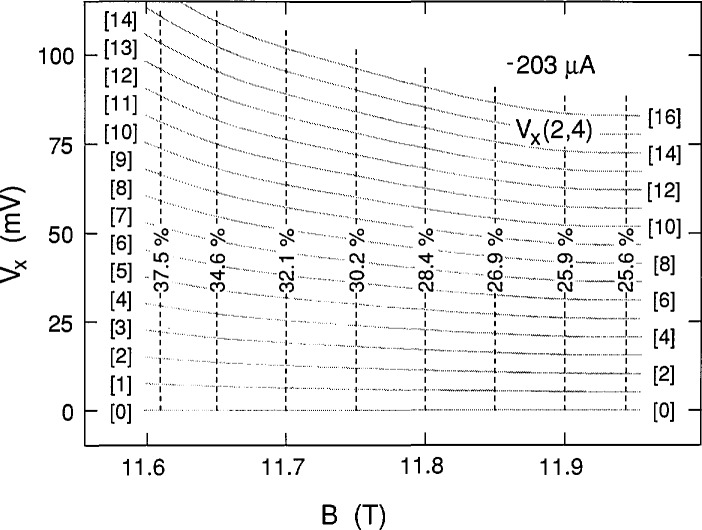
The fractions *f* for a family of curves used to fit the data obtained at −203 μA. This family is slightly different from the one displayed in Fig. 8.

**Fig. 14:**
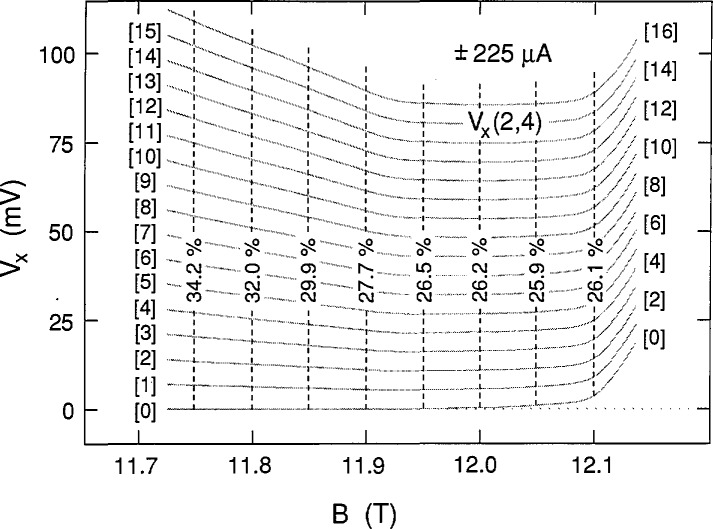
The fractions *f* for the 17 shaded curves shown in Fig. 11 at ±225 μA.

**Fig. 15:**
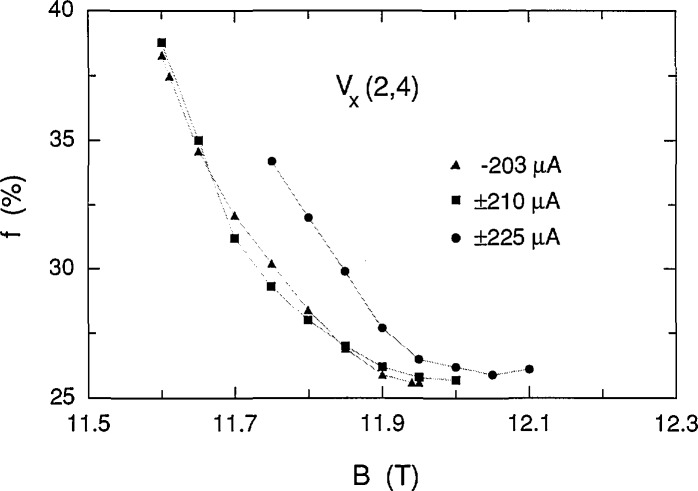
The combined results of *f* versus *B* from Figs. 12–14 for the three currents investigated. The values of *f* have an accuracy of ~1% and a resolution of ~0.1%.

The family of shaded curves in Fig. 8 is the same as that in Fig. 4. Therefore the values of *f* obtained at 203 μA are the same as those shown in Fig. 12 at 210 μA. An independent family of curves was also fitted to the data of Fig. 8. The resulting values of *f* for the independent family are displayed in Fig. 13. They differ from those in Fig. 12 by as much as 0.9%, indicating that *f* can be determined to a precision of about 0.1% and an accuracy of about 1% for these particular data. Figure 14 shows values of *f* for the data of Fig. 11 at 225 μA. The results of *f* versus *B* from Figs. 12–14 are combined in Fig. 15 for the three currents investigated. The difference between the *f* versus *B* curves for −203 μA and ±210 μA in Fig. 15 illustrates the 1% accuracy at which the values of *f* can be determined for these data since they both yielded good fits to the *V_x_* versus *B* curves at −203 μA. The minimum value of *f* at 225 μA is essentially the same as at 203 and 210 μA; however, at lower magnetic field values, *f* is larger at this higher current.

### 3.4 Discussion

The fraction *f* of conducting electrons that make the transitions can be quite large. This suggests that either, all the current enters the breakdown region (in which case *f* is the probability for single transitions), or that some of the current bypasses the breakdown region (in which case *f* would correspond to the fraction of current passing through the breakdown region if the transition probability was always 100%).

The fraction *f* is not necessarily 100%, and, in general, is a function of *B* and *I*. These facts can greatly complicate the identification of voltage quantization for most breakdown data because the voltage separations will not be constant if *f* and *r* are not constant across the magnetic field range, so the voltages will appear to not be quantized even when they actually are.

One can always obtain the *product fM* from the data by using Eq. (1), but the value of *f* can only be determined if *M* can be unambiguously deduced. The data presented here are particularly striking and clear, with sharp vertical transitions, switching between states, and sufficient variations between sweeps to generate the families of shaded curves. Although time-consuming, it was thus relatively easy to determine the quantization. We can therefore be reasonably assured that the values of *M*, and thereby the values of *f*, have been properly determined. Most breakdown data, however, require very careful measurements to deduce the quantization, and in many cases there may be insufficient structure, switching, and variation to definitively determine *M*.

## 4. Conclusions

Quantized dissipative voltage states exist in the breakdown regime of the quantum Hall effect. This quantization has been interpreted using a simple model in which electrons make transitions consisting of excitations from a lower Landau level to a higher level and then a return to the lower level. Voltage quantization suggests that individual electrons either make a single transition or make a fixed number of multiple transitions because varying numbers of transitions would result in a continuum of *V_x_* values rather than voltage quantization.

We have demonstrated that the dissipative voltage states are quantized, and that, in general, the quantization is a function of magnetic field and current. The actual transition mechanisms are no doubt very complicated, so the breakdown region has been treated as a black box, and we used a simple model to interpret the data.

One normally expects quantization phenomena to be predictable, whereas the values of *V_x_* and *f* are not predictable in the present experiment unless the transition probability is actually always 100% and *f* is thus the fraction of current passing through the breakdown region. The quantization is not perfect, but it is surprising just how well quantized the dissipative voltage states are, up to at least the nineteenth excited state.
